# Self-ordered TiO_2 _quantum dot array prepared via anodic oxidation

**DOI:** 10.1186/1556-276X-7-123

**Published:** 2012-02-14

**Authors:** Jana Drbohlavova, Marina Vorozhtsova, Radim Hrdy, Rene Kizek, Ota Salyk, Jaromir Hubalek

**Affiliations:** 1 Department of Microelectronics, Faculty of Electrical Engineering and Communication, Brno University of Technology, Technická 10, Brno 61600, Czech Republic; 2 Department of Chemistry and Biochemistry, Faculty of Agronomy, Mendel University in Brno, Zemědělská 1, Brno 61300, Czech Republic; 3 Institute of Physical and Applied Chemistry, Faculty of Chemistry, Brno University of Technology, Brno 61200, Czech Republic; 4 Central European Institute of Technology, Brno University of Technology, Technická 10, 61600 Brno, Czech Republic

**Keywords:** quantum dots, biosensing, TiO_2_, template methods, nanoporous mask

## Abstract

The template-based methods belong to low-cost and rapid preparation techniques for various nanostructures like nanowires, nanotubes, and nanodots or even quantum dots [QDs]. The nanostructured surfaces with QDs are very promising in the application as a sensor array, also called 'fluorescence array detector.' In particular, this new sensing approach is suitable for the detection of various biomolecules (DNA, proteins) *in vitro *(in clinical diagnostics) as well as for *in vivo *imaging.

The paper deals with the fabrication of TiO_2 _planar nanostructures (QDs) by the process of titanium anodic oxidation through an alumina nanoporous template on a silicon substrate. Scanning electron microscopy observation showed that the average diameter of TiO_2 _QDs is less than 10 nm. Raman spectroscopic characterization of self-organized titania QDs confirmed the presence of an anatase phase after annealing at 400°C in vacuum. Such heat-treated TiO_2 _QDs revealed a broad emission peak in the visible range (characterized by fluorescence spectroscopy).

## Background

Semiconductor quantum dots [QDs] with exceptional physical and optical properties are favorable fluorescent markers in medicine, where they can serve as biosensors and labels in biological imaging [1-3]. Commonly, QDs are used in colloidal form (in aqueous solution) [4,5]. Nowadays, there is a demand for QDs deposited on various solid supports. Nevertheless, directly grown QDs in planar form (so-called lab-on-chip) for *in situ *biosensing purposes are studied very rarely [6]. Occasionally, the scientists try to encapsulate the colloidal QDs in some matrix, e.g., from polymeric compounds [7].

The deposited nanostructures are mostly fabricated through traditional top-down patterning methods like epitaxy or lithographic techniques (mainly photolithography and e*-*beam lithography), which are expensive and time-consuming. Contrary to these methods, the template-based technique seems to be more convenient for nanostructured material synthesis since it is affordable and provides reproducible results. Concerning the template, nanoporous alumina belongs to the most extensively studied materials [8].

The biocompatibility of QDs used for medicinal purposes is usually a problematic issue because most of them are toxic (due to the cadmium ion content), which poses a potential danger especially for future medical applications. Using QDs prepared from titanium dioxide prevents this difficulty because TiO_2 _is a non-toxic material. Nevertheless, there are no scientific papers about the preparation and application of deposited TiO_2 _QDs (only nanodots without the quantum confinement effect). Final quantum confinement of TiO_2 _QDs strongly depends on the anatase/rutile phase composition [9,10]. The phase development of TiO_2 _nanodots is much different from that of TiO_2 _thin films and powders. Generally, the nanodots are polycrystalline and consist of a mixed phase of anatase and rutile after high temperature annealing. However, Chen et al. revealed that TiO_2 _nanodots with a single anatase phase can be prepared from an epitaxial Al/TiN bilayered film on a sapphire substrate by electrochemical anodization of the TiN layer using a nanoporous anodic alumina film as the template [11].

In this work, we modified the experimental method of Chen et al. for the fabrication of TiO_2 _nanodots to prepare a TiO_2 _QD array on silicon substrate with dimensions required to reach the quantum size effect. The key point in our preparation process was to determine the appropriate anodizing potential and deposition time to ensure the optimal nanostructure dimensions, which is necessary from the quantum confinement point of view, as mentioned above. The choice of optimal electrolyte is also significant in order to avoid the possible contamination of the product. The physical, chemical, and luminescence properties of TiO_2 _QDs are discussed as well.

## Methods

### Chemicals

All chemicals such as sulfuric acid (97%, pro analysi [p.a.]), phosphoric acid (84%, p.a.), isopropanol (99.8%, p.a.), and chromium trioxide (99%, p.a.) were purchased from Penta (Prague, Czech Republic). Deionized water underwent demineralization by reverse osmosis using the instrument Aqua Osmotic 02 (Aqua Osmotic, Tisnov, Czech Republic) and subsequent purification using the Millipore RG system and Milli Q water (18.2 MΩ; Millipore Corp., Billerica, MA, USA).

### Titanium and aluminum layer preparation

Prior to titanium deposition, the wafers were degreased and cleaned in isopropanol, rinsed out with deionized water, and finally treated with plasma. A high-purity titanium layer (99.995%, Safina, Jesenice, Czech Republic) with a thickness of 100 nm was prepared by tetrode sputtering on a 4-inch silicon wafer previously coated with a SiO_2 _layer (prepared by thermal CVD). Subsequently, a high-purity aluminum layer (99.99+%) with a thickness of 1 μm was deposited by thermal evaporation (PVD).

### Anodization process

Ordered arrays of titania QDs were achieved by successive anodization of aluminum and titanium layers using the utility model equipment for electrochemical post-processing deposition fabricated in our laboratory (a detailed description of the tool is reported by Hubalek et al. [12]). Thanks to the different anodizing behavior of these layers, the same electrolyte can be applied during the whole process. A concentration of 3 M sulfuric acid was chosen as electrolyte in a constant potential mode (4 V) at 11°C. This acid provides a smaller pore diameter in the alumina template compared to other commonly used electrolytes (oxalic or phosphoric acids). An aqueous solution of H_3_PO_4 _(50 ml L^-1^) and CrO_3 _(30 g L^-1^) was used for chemical etching of the alumina template (5 min at 60°C).

### Characterization of physical and chemical properties

The size of the QDs was estimated using scanning electron microscopy Mira II MLU (Tescan Mira, Brno, Czech Republic). The topography of the pure Ti layer was analyzed by atomic force microscopy [AFM] (Agilent 5500, Agilent Technologies, Santa Clara, CA, USA) with a 10-nm SiC tip. The phase composition of TiO_2 _QDs was characterized by Raman spectroscopy (Renishaw, Wotton-under-Edge, UK) with a NIR laser operating at 745 nm.

## Results and discussion

**Figure 1:**
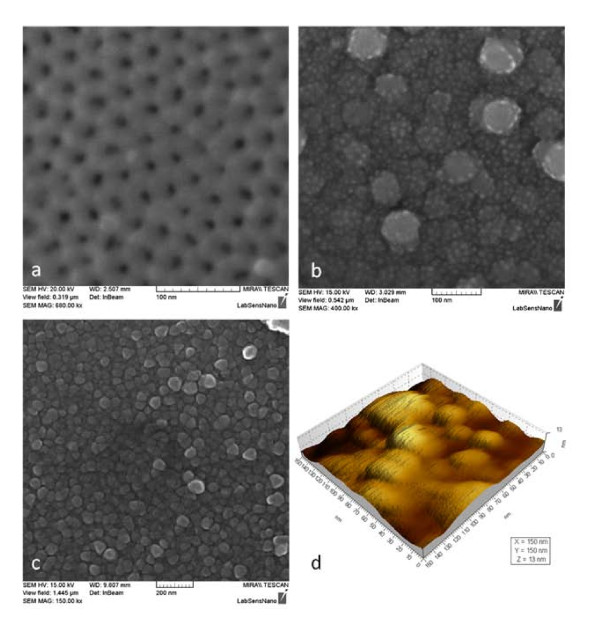
**SEM and AFM images**. SEM characterization of the (**a**) alumina template, (**b**) TiO_2 _QDs on Ti grains, and (**c**) Ti layer. (**d**) AFM image of the Ti layer.

The array of TiO_2 _QDs for biosensing application was prepared through a one-step anodization technique using a nanoporous alumina template. The choice of optimal anodization conditions (4 V, 11°C, and 3 M sulfuric acid as electrolyte) resulted in a template pore size in the range of 5 to 8 nm, which enabled the formation of TiO_2 _QDs with dimensions of about 8 to 10 nm (confirmed by SEM). According to Monticone et al., this dimension of TiO_2 _QDs seems to be appropriate to reach the quantum size effect because they observed almost no variation of the band gap energy with the particle size 2R down to 15 nm [13]. The SEM characterization of the alumina template and TiO_2 _QDs provided important information about QD ordering and homogeneity (see Figure 1a, b, respectively). The pores as well as the QDs were hexagonally arranged as we expected. The interpore spacing in the template was estimated at approximately 15 nm. Figure 1c clearly shows Ti grains in the sputtered titanium layer with a thickness of 100 nm before aluminum layer deposition. These titanium grains are also visible under TiO_2 _QDs as can be seen on Figure 1b. The titanium surface was also analyzed by AFM with a 10-nm tip (see Figure 1d), but this method was not as accurate as the SEM characterization in our case. Particularly, AFM shows the titanium grains as having overlapped and merged structures. From this reason, AFM with A 10-nm tip is not appropriate for the characterization of our sample with TiO_2 _QDs.

**Figure 2:**
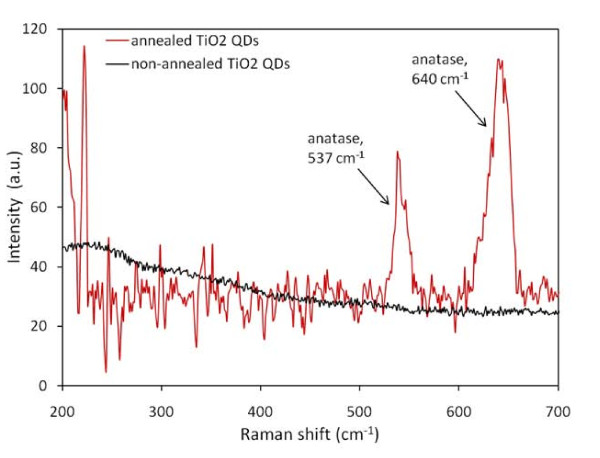
**Raman spectra of annealed and non-annealed TiO_2 _QDs**.

Annealing of QDs at 400°C after alumina template dissolution in the mixture of CrO_3 _and H_3_PO_4 _resulted in the transformation of amorphous titanium dioxide into a crystallographic form. The crystallographic phase composition was characterized by Raman spectroscopy (see Figure 2). The Raman spectrum shows two characteristic anatase peaks: the one at 537 cm^-1^, which is typically presented at 520 cm^-1 ^(corresponding to the A_1g _and B_1g _vibrational mode) and the other at 640 cm^-1 ^(corresponding to the E_g _vibrational mode). Generally, Raman spectra can be affected by the chemical and structural inhomogeneities, which cause the shifts in the corresponding position of Raman bands [14]. Some modifications in band position can be also attributed to the reduction in TiO_2 _particle size, phonon confinement, and oxygen deficiency. From the chemical purity point of view, the sample with TiO_2 _QDs was characterized by EDX, which confirmed the presence of the Ti, Si, and O elements corresponding to TiO_2 _QDs, Ti and SiO_2 _layers, and Si wafer.

**Figure 3:**
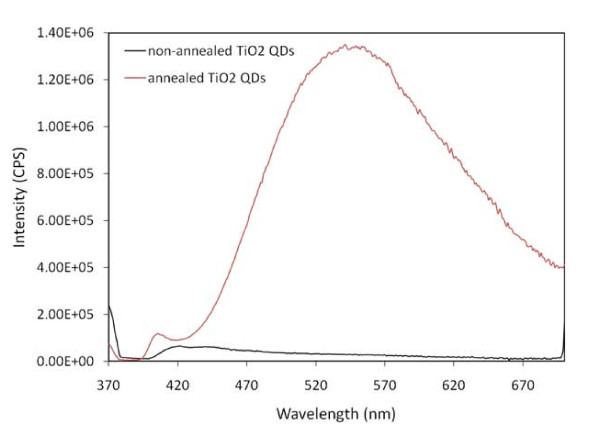
**Fluorescence spectra of annealed and non-annealed TiO_2 _QDs**.

The photoluminescence properties of the prepared TiO_2 _QDs array were characterized by fluorescence spectroscopy. Figure 3 representing the fluorescent spectrum of annealed the TiO_2 _QDs shows a broad emission peak in the range of 440 nm up to 580 nm, while the spectrum of the as-prepared TiO_2 _QDs without annealing shows no peaks, which means that this sample is composed predominantly from amorphous TiO_2_.

## Conclusion

The cheap, rapid, and easy reproducible template-based method was used successfully for the synthesis of titania QD sensor array suitable for various biomolecule (DNA, proteins) sensing *in vitro*. The anodization process through the highly ordered alumina nanoporous template provided less than 10-nm-sized TiO_2 _QDs densely covering the titanium surface and showing the strong photoluminescence peak in the visible range. The usage of this fluorescence array detector may be of great importance for clinical diagnostics and *in vivo *imaging.

## Competing interests

The authors declare that they have no competing interests.

## Authors' contributions

JD carried out the fabrication of titania QDs and their characterization using Raman and fluorescence spectroscopies. MV participated in the fabrication of QDs array. RH carried out the SEM characterization. RK drafted the manuscript. OS carried out the deposition of metalic layers. JH participated in the design of the fluorescence array detector. All authors read and approved the final manuscript.
